# Dysregulations in the PI3K pathway and targeted therapies for head and neck squamous cell carcinoma

**DOI:** 10.18632/oncotarget.14729

**Published:** 2017-01-18

**Authors:** Yi Cai, Sonam Dodhia, Gloria H Su

**Affiliations:** ^1^ Department of Otolaryngology-Head and Neck Surgery, Columbia University Medical Center, New York, NY, USA; ^2^ Herbert Irving Comprehensive Cancer Center, Columbia University Medical Center, New York, NY, USA; ^3^ Department of Pathology and Cell Biology, Columbia University Medical Center, New York, NY, USA

**Keywords:** PI3K signaling, head and neck squamous cell carcinoma, PIK3CA mutation, PI3K inhibitor, personalized medicine

## Abstract

The phosphoinositide 3-kinase (PI3K) signaling pathway is the most commonly mutated pathway in head and neck squamous cell carcinoma (HNSCC). There are several drugs targeting members of the PI3K signaling pathway in development for HNSCC. In this article, we review the genetic alterations reported in the pathway pertinent to HNSCC, various agents in development targeting various mediators of the pathway, results from clinical trials, and remaining challenges in the development of PI3K pathway inhibitors.

## INTRODUCTION

The phosphoinositide 3-kinase (PI3K) signaling pathway is of particular importance in head and neck squamous cell carcinoma (HNSCC), as it is the most frequently mutated pathway [1, 2]. Under normal conditions, this signaling pathway serves to promote cell survival, growth, development, and differentiation [3, 4]. Recently, dysregulation of this pathway has been noted at the genomic and proteomic levels, with implications in both the pathogenesis of HNSCC and potential therapeutic targets. In addition, activation of the PI3K pathway is known to be involved in acquired resistance to anticancer therapy [5]. In this review, we discuss the dysregulation of the PI3K signaling pathway, target therapies, and implications for precision medicine in the context of HNSCC.

## PI3K SIGNALING PATHWAY IN NORMAL PHYSIOLOGY

Key players of the PI3K pathway include receptor tyrosine kinases (RTKs) such as epidermal growth factor receptor (EGFR), G-protein coupled receptors (GPCRs), PI3Ks, phosphatidylinositol 4,5-bisphosphate (PIP_2_), phosphatidylinositol (3,4,5)-trisphosphate (PIP_3_), Akt, mTOR, and PTEN. RTKs and GPCRs bind growth factors and cytokines at the cell surface, and then transduce signals via a number of intracellular pathways, such as the PI3K pathway. PI3Ks are classified into three groups: classes I, II, and III. Class I is subdivided into class IA and class IB and they consist of a catalytic (p110) and regulatory (p85 for class IA and p101 and p87 for class IB) components. Class IA is activated by various types of cell surface receptors and has 3 isoforms, p110α, p110β, and p110δ. Class IB is activated primarily by G-protein coupled receptors and has one isoform, p110γ. Of these isoforms, class IA PI3Ks have been studied the most extensively. Binding of p85 to p110 stabilizes p110, inhibits their kinase activity, and recruits phosphotyrosine in activated receptors or adaptors. The engagement of p85 and phosphotyrosine activates the kinase activity of p110 by releasing the p85-mediated inhibition of p110 [6]. The activated catalytic subunit of PI3K (p110) phosphorylates PIP_2_, generating PIP_3_. PIP_3_ binds Akt (protein kinase B), a serine/threonine protein kinase, leading to the exposure of two amino acid residues requiring phosphorylation. PIP_3_ also binds phosphoinositide-dependent protein kinase-1 (PDK1), which phosphorylates those two residues, leading to the full activation of Akt. Activated Akt then phosphorylates other proteins and thus affects a number of cellular processes. mTOR, another serine threonine protein kinase, is activated by Akt and plays an important role in cell growth by monitoring cellular needs, such as nutrients, oxygen, and energy. mTOR forms two distinct complexes with other proteins and both complexes are involved in tumorigenesis [7, 8]. Akt activates mTORC1 indirectly by inactivating proline-rich Akt substrate 40 kDa (PRAS40) and tuberous sclerosis complex (TSC2), which inhibit mTORC1. mTORC1 then regulates protein translation, specifically by targeting ribosomal protein S6 (pS6) kinase and eukaryote initiation factor 4E-binding protein 1 (4E-BP1). mTOR complex 2 (mTORC2) is involved in activation of Akt by phosphorylation of Ser473 in Akt. The mechanism of mTORC2 activation is not clear. Lastly, PTEN (phosphatase and tensin homology deleted from chromosome 10) is an important negative regulator of this pathway, as it serves to dephosphorylate PIP_3_ to PIP_2_ [9, 10].

## PI3K SIGNALING PATHWAY IN HNSCC

Recent studies have worked to identify mutations, amplifications, and overexpression of the different mediators/genes involved in the PI3K pathway (Table 1). Of these, *PIK3CA*, the gene that encodes for the catalytic component p110α, is the most commonly mutated component of this pathway.

**Table 1:** Dysregulations in the PI3K pathway in HNSCC

PI3K Pathway Component	Type of alteration	Tumor site
p85	Mutation [2, 11, 12]	
PTEN	Mutation [2, 13–15] Loss of heterozygosity [16, 17] Reduced expression [16]	Not specified [2, 13, 14] Oropharynx, hypopharynx, larynx [15] Tongue, larynx, oral cavity [17] Not specified [16]
AKT	Mutation [2] Activation [16, 18] Copy number alteration [19] Over-expression [20]	
mTORC1	Mutation [2] Activation [21]	
EGFR	Mutation [22, 23] Activation [24] Overexpression [24]	Laryngeal [23] Not specified [22]

In cases where tumor sites were not specified, the table entry was left blank. Few studies [2, 11, 12] specified HPV status.

## PIK3CA Mutations

**Figure 1:**
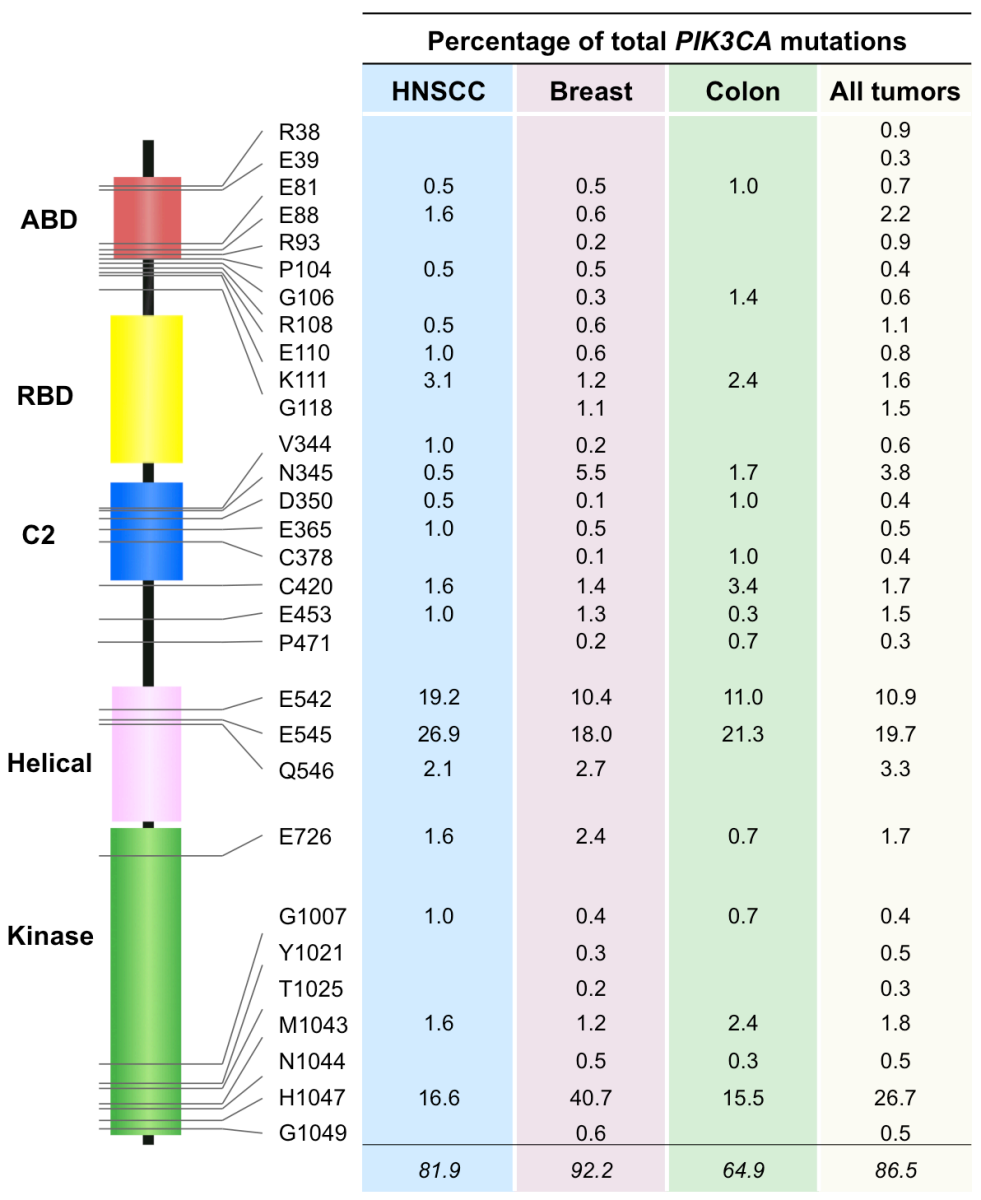
PIK3CA point mutation rates in TCGA cohorts Analysis of TCGA cohorts of HNSCC, breast cancer, and colorectal cancer was performed to determine rates of point mutations in *PIK3CA*. The top 30 most frequent mutations when analyzing all TCGA tumor samples were selected. The residues affected by these mutations are depicted along with the corresponding rates of mutation. The mutation rates represent the percentage of total *PIK3CA* mutations affecting each residue for the various types of tumor. Abbreviations: ABD, adaptor-binding domain; RBD, RAS-binding domain; C2, protein-kinase-C homology-2 domain.

In HNSCC, *PIK3CA* mutations tend to be heavily focused on the helicase (exon 9) and kinase (exon 20) domains [25–27], which also holds true for *PIK3CA* mutations in most sporadic cancers (Figure 1). In the past few years, genomic datasets of HNSCCs have expanded with efforts led by The Cancer Genome Atlas (TCGA) [28, 29]. In Figure 1, the *PIK3CA* point mutation frequencies of HNSCC, breast cancer, and colorectal cancers catalogued by the TCGA are compared. The frequencies of *PIK3CA* mutations were 17.5%, 36.4%, and 16.7% in HNSCC, breast, and colorectal cancer, respectively. Of note, the hot-spot mutations on exon 9 (corresponding to residues E542 and E545 in p110α) and exon 20 (residue H1047) are the most common alterations regardless of cancer type. E542 and E545 are frequently mutated to lysine and H1047 is frequently altered to arginine.

*PIK3CA* over-amplification [16, 20, 30] and copy number gain [20, 31] have also been reported in HNSCC. Moreover, *PIK3CA* overexpression and copy number gains in the PI3K pathway have been associated with HPV-positive HNSCC [32, 33]. Though copy number gains have been linked to increased transcription and expression of p110α [34], there are not necessarily correlations between *PIK3CA* gene amplification and p110α expression [16]. This suggests that there may be other pathways that interact with or influence p110α expression and play a role in tumorigenesis.

## MUTATIONS IN OTHER COMPONENTS OF THE PI3K PATHWAY

p85, the regulatory subunit of Class IA PI3K, has three isoforms — p85α, p85β, and p55γ — that are encoded by the genes *PIK3R1*, *PIK3R2*, and *PIK3R3*, respectively. Mutants of p85 are oncogenic, as they have been shown to promote basal PI3K activity without stimulation by growth factors [35–37]. In HNSCC, *PIK3R1* missense [12], in-frame insertion [12], nonsense [2], and non-synonymous [11] mutations have been noted, mostly in HPV-negative patients. Depending on whether p85 exists as a monomer or a dimer with p110, it plays different roles. As a monomer, it binds the cell surface receptor adaptor protein IRS-1 and limits downstream PI3K pathway signaling. As a dimer with p110, however, it serves to potentiate PI3K signaling. If a gene such as *PIK3R1*, which encodes for a component of p85, is dysfunctional, adequate p85 may not be produced, leading to hyperactivation of the PI3K pathway and resultant tumorigenesis [35]. In addition, *PIK3R2* overexpression was noted in esophageal squamous cell carcinoma, and is thought to be related to under-expression of microRNA-126, which likely plays a tumor-suppressing role and targets *PIK3R2* [38].

PTEN is a tumor suppressor that has been found to have a number of alterations in HNSCC, including nonsense [2, 13, 14], missense [2, 13, 15], loss of heterozygosity [16, 17], hemizygous deletion [39], intron [14], and splice site single nucleotide polymorphisms [2], as well as reduced expression [16]. Given the many different genomic and proteomic alterations seen with PTEN, its dysregulation in HNSCC might be attributed to multiple molecular mechanisms. With regard to genetic mutations, exon 5 of the *PTEN* gene is of particular interest, as it encodes for PTEN's lipid phosphatase catalytic domain, which plays a major role in tumor suppression [40]. Low PTEN protein expression (not necessarily linked to *PTEN* gene mutations) may be attributed to degradation of PTEN [41, 42] or gene silencing [43, 44].

Akt promotes cell survival and proliferation through the phosphorylation of various substrates and is encoded by 3 Akt genes (*Akt1*, *Akt2*, and *Akt3*) [45]. In HNSCC, Akt genes have been found to display missense mutations [2], copy number variations [19], increased activation [16, 18, 46], and overexpression [20]. Amplification and overexpression of Akt is thought to enable a cell to proliferate in conditions not normally conducive to proliferation [47]. mTOR is a downstream target of Akt that integrates signals from multiple pathways, including nutrients (e.g., amino acids and glucose), growth factors (e.g., insulin and insulin-like growth factor 1), hormones (e.g., leptin), and stresses (e.g., starvation, hypoxia, and DNA damage) to regulate a wide variety of eukaryotic cellular functions, such as translation, transcription, protein turnover, cell growth, differentiation, cell survival, metabolism, energy balance, and stress response. In HNSCC, mTOR was found to be activated [21, 48] and have missense mutations [2]. It has been demonstrated that these point mutations can lead to constitutive activation of mTOR, which enables cells to grow and proliferate in the absence of nutrients [49].

EGFR is an upstream activator of the PI3K signaling pathway that is frequently altered in cancer. It is a member of the RTK (receptor tyrosine kinases) that activates class IA PI3Ks, the most commonly mutated members of the PI3K pathway in cancer. It has been reported to display missense [23], in-frame deletion [22], and activating mutations [24], as well as overexpression [24] in HNSCC. An abnormally truncated EGFR mutant (deletion in exons 2-7) has been found to be constitutively active and potentiate the PI3K pathway signaling [50], and may play a role in tumorigenesis.

### Clinical trials targeting the PI3K pathway in HNSCC

**Figure 2:**
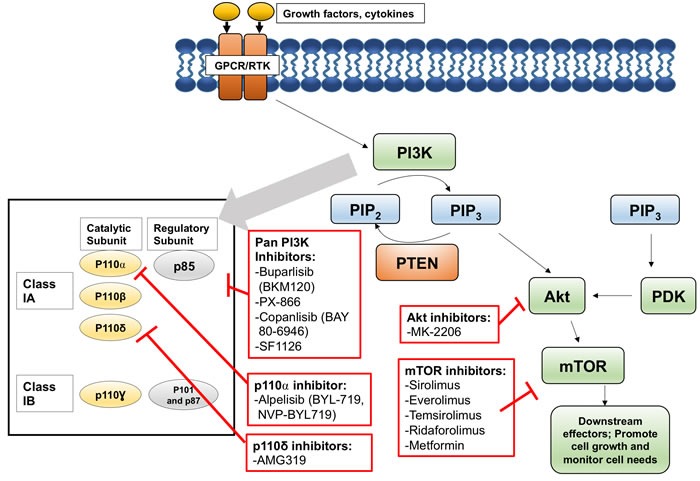
The PI3K signaling pathway's key players and inhibitors under investigation in HNSCC trials

Treatment modalities for HNSCC are guided by site and stage of disease and include surgical resection, radiation, and chemotherapy. However, when tumors have developed resistance, relapse occurs and these treatments are no longer effective and new therapeutic options are needed. The PI3K/Akt/mTOR pathway is activated in many types of cancers and has been demonstrated to contribute to treatment resistance [5]. In addition, this pathway has been implicated in tumorigenic processes such as cell proliferation, invasion, angiogenesis, and metastasis [45, 51–53]. These factors make the PI3K/Akt/mTOR pathway an attractive target for cancer therapy. Significant efforts have been devoted to developing agents to target the pathway [54–56]. Many of these agents have shown promising results in preclinical *in vitro* and *in vivo* studies of various cancer types [57, 58], including HNSCC [59, 60]. Inhibition of this pathway can disrupt resistance acquired by cancer cells and sensitize cancers to antitumor agents of other modalities, reduce cell proliferation, and induce apoptosis [5, 61–63]. Furthermore, single inhibitors may not be enough to achieve sustained inhibition of the pathway. It has been shown that inhibition of PI3K pathway may trigger compensatory feedback [64]. Therefore, in the majority of the clinical trials, PI3K/Akt/mTOR inhibitors are used in combination with other agents or radiation with the goal of achieving a synergistic effect [65]. Here, we discuss the recent development of PI3K pathway inhibitors that have been or are being tested in clinical trials for HNSCC (Figure 2). The relevant clinical trials registered at ClinicalTrials.gov are listed in Table 2.

**Table 2:** Ongoing Clinical Trials of Agents Targeting the PI3K Pathway in Patients with HNSCC

Target	Agent	Other Interventions	Condition	Phase	Status	Clinical Trial Identifier
PI3K	Buparlisib (BKM120)		Advanced HNSCC	2	Unknown	NCT01527877
		Cisplatin, IMRT	High risk LA HNSCC	1b	Active	NCT02113878
		Paclitaxel	Pt pre-treated R/M HNSCC	2	Active	NCT01852292
		Cetuximab	R/M HNSCC	1/2	Active	NCT01816984
			R/M HNC	2	Active	NCT01737450
	PX-866	Docetaxel	NSCLC, HNSCC	1/2	Completed	NCT01204099
		Cetuximab	Metastatic CRC, R/M HNSCC	1/2	Completed	NCT01252628
	Copanlisib (BAY 80-6946)	Cetuximab	R/M HNSCC with PI3KCA mutation/amplification and/or PTEN loss	1/2	Active	NCT02822482
	SF1126		R/M SCCHN with mutation in PIK3CA and/or PI3K Pathway	2	Active	NCT02644122
	Alpelisib (BYL-719)	Cisplatin, IMRT	LA HNSCC	1	Active	NCT02537223
		Paclitaxel	Breast cancer and HNC	1	Active	NCT02051751
		Cetuximab, IMRT	Stage III/IVb HNSCC	1	Active	NCT02282371
			Pt therapy failed, R/M HNSCC	2	Active	NCT02145312
		Cetuximab	R/M HNSCC	1b/2	Completed	NCT01602315
	AMG319		HPV negative HNSCC	2	Active	NCT02540928
AKT	MK2206		R/M HNC	2	Completed	NCT01349933
mTOR	Sirolimus (rapamycin)		HNSCC	1/2	Completed	NCT01195922
	Everolimus (RAD001)	Docetaxel	LA and R/M HNSCC	1/2	Terminated	NCT01313390
		Carboplatin, cetuximab	Advanced HNC	1/2	Completed	NCT01283334
		Carboplatin, paclitaxel	LA HNC not removable by surgery	1/2	Completed	NCT01333085
		Erlotinib	Recurrent HNSCC	2	Completed	NCT00942734
		Erlotinib, radiation	R/M HNSCC treated with radiation	1	Withdrawn	NCT01332279
		Cetuximab	R/M colon cancer or HNC	1	Completed	NCT01637194
		Cetuximab, cisplatin, carboplatin	R/M HNSCC	1/2	Terminated	NCT01009346
			HNSCC	2	Active	NCT01133678
			HNSCC	2	Active	NCT01051791
			HNC	2	Active	NCT01111058
		Docetaxel, cisplatin	LA HNC	1	Completed	NCT00935961
		Cisplatin, radiation	LA, inoperable HNC	1	Terminated	NCT01057277
		Cisplatin, IMRT	LA HNC	1	Terminated	NCT01058408
		Cisplatin, IMRT	LA HNC	1	Completed	NCT00858663
		Ceritinib	HNC, NSCLC	1/1b	Active	NCT02321501
			LA HNSCC	2	Active	NCT01133678
	Temsirolimus (CCI-779)		HNSCC	2	Completed	NCT01172769
		Cetuximab	R/M HNC not respond to therapy	2	Completed	NCT01256385
		Paclitaxel, carboplatin	R/M HNSCC	1/2	Active	NCT01016769
		Cisplatin, cetuximab	R/M HNSCC	1/2	Terminated	NCT01015664
			Advanced HNSCC		Completed	NCT00195299
		Erlotinib	Pt-refractory or-ineligible, advanced SCC	2	Terminated	NCT01009203
		Cetuximab, cisplatin, radiation	Advanced HNC	pilot	Withdrawn	NCT01326468
	Ridaforolimus (AP23573, MK-8669, deforolimus)		Advanced HNC, NSCLC, colon cancer	1	Terminated	NCT01212627
	Metformin (glucophage)	Paclitaxel	R/M HNSCC	2	Terminated	NCT01333852
		Cisplatin, radiation	LA HNSCC	1	Active	NCT02325401
			HNSCC	0	Active	NCT02402348
			HNSCC	0	Active	NCT02083692
PI3K/AKT	Perifosine (KRX-0401)		R/M HNC	2	Terminated	NCT00062387
PI3K/PLK	Rigosertib (ON-01910)	Cisplatin, radiation	HNSCC	1	Completed	NCT02107235

Unknown: Status not verified over 2 years. Abbreviations: IMRT: Intensity-modulated radiotherapy. HNC: Head and Neck Cancer. SCC: Squamous cell carcinoma. HNSCC: Head and neck squamous cell carcinoma. NSCLC: Non small cell lung cancer. HPV: Human Papillomavirus. R/M: recurrent and/or metastatic

## PAN-PI3K INHIBITORS

Pan-PI3K inhibitors refer to inhibitors of more than one of the p110 isoforms of PI3K.

### Buparlisib (BKM120)

Buparlisib is an oral PI3K inhibitor and inhibits the activity of all four p110 isoforms of class I PI3K. Using cultured cell lines with wild-type or E542K, E545K, and H1047R hotspot mutations in *PIK3CA*, Kong *et al*. showed that buparlisib has comparable impact on the hotspot and wild-type *PIK3CA* [66]. It was also shown that a combination of buparlisib with the anti-EGFR monoclonal antibody cetuximab exerts a synergistic effect on tumor inhibition in wild-type or *PIK3CA* mutant HNSCC cell lines [67] as well as in a xenograft model of HNSCC [68]. However, the half maximal inhibitory concentration (IC_50_) of buparlisib is much higher than that of many of the PI3K inhibitors under investigation. The toxicity of doses required for PI3K inhibition *in vivo* is a concern in its clinical application. Five phase 1 and/or 2 clinical trials are ongoing to evaluate the efficacy and safety of buparlisib in combination with cisplatin and IMRT, paclitaxel, or cetuximab (Table 2). Results from these trials have not been posted.

### PX-866

Wortmannin is a potent irreversible PI3K inhibitor that equally inhibits most PI3K isoforms and has been shown to have antitumor activity. However, it is not optimal for clinical applications due to its high liver and hematological toxicity as well as poor biological stability. PX-866 was derived from wortmannin to overcome these problems [69]. It can inhibit PI3K activity at 0.1 nM and was reported to enhance antitumor activity of other chemotherapeutic drugs and radiation in an ovarian cancer murine xenograft model [69]. However, phase 1 and 2 clinical trials of PX-866 in incurable recurrent or metastatic HNSCC patients have shown less promise. For example, a phase 2 randomized control trial using combination therapy with cetuximab showed no differences in disease control rate, median progression-free survival, or median overall survival when compared to the control group receiving cetuximab alone [70]. A phase 2 randomized control trial using combination therapy with docetaxel also showed no differences when compared to the control group receiving cetuximab [71]. Thus, the addition of PX-866 did not improve the efficacy in patients without molecular preselection. No active trial of PX-866 for HNSCC is currently registered.

### Copanlisib (BAY 80-6946)

Copanlisib is a highly selective and potent intravenous pan-PI3K inhibitor with sub-nanomolar IC_50_ against isoforms p110α and p110δ [72]. The mean IC_50_s of copanlisib were 19 nM and 774 nM for *PIK3CA* mutant and wild type cell lines respectively, indicating superior antitumor activity. In non-small cell lung cancer xenograft models, combination of copanlisib and paclitaxel achieved a 100% sustained response. Copanlisib in combination with cetuximab is being evaluated in phase 1 and phase 2 trials in patients with recurrent and/or metastatic HNSCC harboring a *PI3KCA* mutation/amplification and/or a *PTEN* loss.

### SF1126

LY294002 can inhibit both PI3K and mTOR but is generally considered a PI3K inhibitor. It has antitumor and antiangiogenesis activity *in vivo*, but is not a viable drug due to poor solubility and short half-life. SF1126 was designed as a prodrug of LY294002 with a small peptide tag on LY294002 to increase solubility and to target αvβ3 and α5β1 integrins [73]. SF1126 was able to significantly reduce tumor volumes in U87MG glioma and PC-3 prostate cancer xenograft models in nude mice. In addition, antiangiogenesis activity due to inhibition of the HIF-1/VEGF pathway activity in 6 of 11 xenograft models was observed. SF1126 is now being evaluated in a phase 2 trial in patients with recurrent or progressive HNSCC and mutations in *PIK3CA* and/or PI3K pathway genes.

## ISOFORM-SPECIFIC PI3K INHIBITORS

Isoform-specific PI3K inhibitors are active against one of the p110 isoforms of class I PI3K. They are usually also active against other p110 isoforms to a lesser extent.

### Alpelisib (BYL-719, NVP-BYL719)

Alpelisib was designed to selectively inhibit p110α, or *PIK3CA* [74]. In a *PIK3CA*-dependent murine xenograft model, alpelisib showed significant dose-dependent inhibition of tumor growth and a favorable safety profile [75]. These results suggest that alpelisib is a promising agent for treating tumors with *PIK3CA* mutations. Alpelisib is being evaluated in 5 clinical trials (Table 2). Preliminary results showed encouraging antitumor activity [76]. Results from a completed trial have not been posted.

### Isoform p110δ of PI3K (PI3Kδ, AMG319)

p110δ is mostly confined to spleen, thymus, and peripheral blood leukocytes. Its dysregulation has been implicated in rheumatoid arthritis, systemic lupus erythematosus, and hematological malignancies. Inactivation of p110δ in regulatory T cells unleashes CD8^+^ cytotoxic T cells and induces tumor regression. Thus, p110δ inhibitors such as AMG319 can break tumor-induced immune tolerance [77]. Currently, AMG319 is in a double-blind, placebo-controlled phase 2a trial in patients with HPV-negative HNSCC.

## AKT INHIBITORS

### MK-2206

Akt activation and overexpression are often associated with resistance to chemotherapy or radiotherapy. Inhibition of Akt has great potential in cancer treatment. Many Akt inhibitors have been developed for cancer treatment [55]. Among them, MK-2206 is a highly potent and selective Akt inhibitor that has been shown to enhance the anti-tumor activity of several anticancer agents *in vitro* and *in vivo* [78]. MK-2206 was evaluated in a phase 2 trial in patients with recurrent or metastatic HNSCC. Nine out of 21 patients were alive and progression-free at the end of the trial (but final results have not been posted). Moreover, MK-2206 plus carboplatin/paclitaxel, docetaxel, or erlotinib was evaluated in a phase 1 trial in patients with advanced solid tumors [79]. Interestingly, two patients with HNSCC demonstrated a complete and partial response.

## MTOR INHIBITORS

Rapamycin was isolated from *Streptomyces hygroscopicus*. It was initially developed as an anti-fungal agent and used as an immunosuppressant to prevent rejection in organ transplant. Later, it was found to inhibit mTOR and showed promising antitumor activity in many solid tumors. Rapamycin binds to the intracellular FK506-binding protein (FKBP12) to form a complex, which then binds to mTORC1 and interrupts its ability to signal to its downstream effectors [56, 80, 81]. Temsirolimus, everolimus, and deferolimus are analogs of rapamycin and they inhibit mTORC1 activity through the same mechanism.

### Sirolimus (rapamycin)

Sirolimus has been tested for treatment of many types of tumors, including HNSCC, but has poor oral bioavailability and solubility (precluding intravenous administration). Analogs of sirolimus have been developed to overcome this problem. In addition, nanoparticle albumin-bound rapamycin has been developed and is currently being tested in a phase 2 trial that includes HNC.

### Everolimus (RAD001)

Everolimus is a 2-hydroxyethyl derivative of sirolimus with a similar mechanism of action, but an improved oral bioavailability. Everolimus has been approved by the U.S. Food and Drug Administration (FDA) for treatment of several cancers including recent approval for the treatment of progressive, well differentiated, nonfunctional, neuroendocrine tumors of gastrointestinal or lung origin in unresectable, locally advanced, or metastatic disease. In a trial with recurrent or metastatic HNSCC patients, everolimus was not effective [82]. Everolimus, in combination with cetuximab/carboplatin, or cisplatin/IMRT, or cisplatin/docetaxel, has been evaluated in several early phase studies [83–85]. In a phase 2 study, everolimus in combination with erlotinib was evaluated in patients with platinum-resistant HNSCC with no significant benefit [86]. Currently, two other phase 2 trials are ongoing to evaluate the efficacy of everolimus in patients with refractory, recurrent, and locally advanced HNSCC and to study the correlation of everolimus treatment with tumor- and patient-associated markers of the EGFR-mTOR pathway.

### Temsirolimus (CCI-779)

Temsirolimus is hydrolyzed to form sirolimus quickly after intravenous administration but itself also has mTOR inhibitor activity. It has been approved by the FDA to treat renal cell carcinoma and evaluated in many trials of combination therapy for HNSCC. In a pharmacodynamic evaluation of temsirolimus in patients with newly diagnosed advanced HNSCC, Akt/mTOR pathway biomarkers were evaluated in tumor and peripheral blood mononuclear cells (PBMCs). Temsirolimus significantly decreased pS6 and p4E-BP1 in tumors, and pS6 and pAkt in PBMCs, indicating significant inhibition of the mTOR pathway in both tumors and PBMCs [87]. In addition, after only 2-3 doses, 8 of 14 patients’ tumors decreased in size on endoscopic evaluation [87]. In a different trial of patients with platinum- and cetuximab-refractory recurrent and/or metastatic HNSCC, treatment with temsirolimus resulted in disease stabilization in 58% and tumor shrinkage in 39% of 33 assessable patients [88]. However, combination therapy with temsirolimus may be limited by treatment toxicity. In trials studying combination therapy of temsirolimus, bevacizumab, and cetuximab or temsirolimus and erlotinib, numerous toxicities were reported [89, 90]. The combination of temsirolimus with weekly does of paclitaxel and carboplatin was evaluated in a phase 1 and 2 trial in patients with recurrent or metastatic HNSCC to establish recommended dosing for a phase 2 study and to determine the objective response rate.

### Ridaforolimus (AP23573, MK-8669, deforolimus)

Ridaforolimus is available in oral and intravenous formulations. In combination with the Notch inhibitor MK-0752, ridaforolimus was evaluated in a phase 1 trial in patients with advanced tumors [91]. Fifteen of 30 enrolled patients had HNSCC. Among 10 HNSCC patients evaluated for tumor response, one had complete response and another had a confirmed partial response. However, there were a high number of adverse events at the maximum tolerated dose.

### Metformin (glucophage)

Metformin is used in the treatment of type 2 diabetes. The use of metformin in diabetic patients has been associated with significantly lower risks of cancer incidence and mortality. In a retrospective study of 205 patients with laryngeal SCC [92], patients treated with metformin had more early stage tumors than untreated patients (48% vs 27%), but had fewer regional metastasis events (19% vs 50%) and a better survival rate (76% vs 41%). Yen *et al*. compared 66,600 diabetic patients either taking or not taking metformin and found the incidence of head and neck cancer (HNC) to be 34% lower in patients taking metformin [93]. Metformin indirectly inhibits mTOR by decreasing expression of Sp-regulated insulin-like growth factor-1 receptor and also inhibits Ras signaling by decreasing EGFR [94]. Skinner *et al*. investigated the role of *TP53*-disruptive mutations in radioresistance [95]. They found metformin potentiated the effects of radiation in the presence of a disruptive *TP53* mutation *in vitro* and *in vivo*. Among patients treated with postoperative radiation therapy for HNSCC, patients taking metformin had a dramatically lower locoregional recurrence rate than did the control group. The five-year overall survival rate was 87% in patients taking metformin compared to 41% in the remaining patients [95]. The safety and efficacy of metformin, alone or in combination with radiation or other agents, are being evaluated in several early stage clinical trials in HNSCC patients (Table 2).

## DUAL INHIBITORS

### Perifosine (D-21266, KRX-0401, NSC 639966)

Perifosine inhibits both protein kinase B and Akt phosphorylation, but does not directly inhibit PI3K [96]. Though it inhibited tumor growth in various *in vitro* and *in vivo* studies [97], it showed little effect in a phase 2 trial of patients with recurrent or metastatic HNC that was terminated early [98]. Perifosine alone or in combination with other agents is currently being tested in several trials for cancers, but not in HNSCC.

### Rigosertib (ON 01910.Na, estybon)

Polo-like kinases (Plks) are important regulators of the cell cycle and are new targets for cancer therapy [99, 100]. Rigosertib was developed to inhibit Plk [101] and was found to inhibit PI3K as well [102]. Rigosertib's antitumor activity was studied in 16 HPV-negative HNSCC cell lines and 8 direct patient tumor xenografts of HNSCC [103]. Rigosertib had potent antiproliferative effects on 11 of 16 HNSCC cell lines and inhibited growth reduction in 3 of 8 HNSCC xenografts. Biomarker analysis indicated that a combination of PI3K/TP53 events was necessary, but not sufficient, for rigosertib sensitivity. Safety and efficacy of rigosertib were evaluated in a recently completed phase 2 trial in patients with relapsed or metastatic, platinum-resistant, HPV-positive or HPV-negative SCC. Results from the trial have not been posted or published.

## REMAINING CHALLENGES

Studies of PI3K pathway inhibitors on HNSCC cell lines and xenograft models have been very encouraging, but these agents have shown less promise in clinical trials to date. Several reasons may explain this discrepancy. As mentioned earlier, single agent therapy may have limited efficacy due to activation of compensatory feedback [64]. PI3K inhibition may also activate mitochondrial reprogramming that subsequently promotes tumor invasion and progression [104]. Yet, beyond unintended molecular signaling that can be induced by PI3K pathway inhibition, there are also challenges in maximizing clinical trial design. For one, non-optimized dosing schedules may lead to inadequate pharmacologic inhibition of the pathway [105].In addition, trial results may also be affected by inadequate patient preselection [57, 58]. Existing HNSCC trials of PI3K pathway inhibitors have stratified patients based on prior treatment failures [70, 71]. However, few published studies have examined trial results for a correlation between responsiveness and PI3K pathway mutational status. Furthermore, none - to our knowledge - have preselected HNSCC patients based on PI3K pathway mutation status, which likely has a significant effect on response to targeted therapies and trial results [58]. Below, we discuss the potential role of genomics in future HNSCC trials.

## APPLYING PRECISION MEDICINE IN HNSCC TRIALS

In the era of genomics, next generation sequencing offers the ability to characterize the mutational profiles of patients on a genome-wide scale and the potential to enhance future trial design. Genomic sequencing can be readily performed on HNSCC tumor specimens. However, tumor specimens may not be available for all patients enrolled in HNSCC trials, would be costly to obtain, and could place patients at risk for procedural complications. Thus, it would be advantageous to utilize a non-invasive source of tumor samples for genetic profiling. “Liquid biopsies” using circulating tumor cells (CTCs) and tumor cell DNA extracted from the peripheral blood for biomarker analysis could provide a potential solution. Cancers are known to release CTCs and cell-free circulating tumor DNA (ctDNA), or cell-free nuclear acids (cfNA), into blood. Recently, their potential as diagnostic and monitoring tools for cancer has been extensively investigated [106, 107]. Combined with next generation sequencing technology, liquid biopsy can be used to personalize HNSCC patient treatments, evaluate tumor mutations throughout treatment, predict treatment response, and potentially elucidate novel biomarkers. Research on liquid biopsies in HNSCC has lagged behind that in more common cancer types, but is increasingly gaining more attention. In an ongoing trial (NCT02822482) of copanlisib with cetuximab in HNSCC patients with PI3K mutation/amplification and/or PTEN loss, mutational profiles of ctDNA at multiple time points will be analyzed to monitor disease progression.

## CONCLUSIONS

Recent studies on HNSCC continue to support the PI3K pathway as a promising target for future HNSCC therapies. Currently, there are multiple targeted therapies against this pathway under investigation. However, trial results to date have yet to show the same degree of efficacy as have been demonstrated in *in vitro* or *in vivo* studies. Several challenges may be limiting trial success. The results from ongoing trials, such as those with patient recruitment based on mutational profiles of the PI3K pathway or comparisons between single and multiple agent therapies, will be eagerly anticipated and may provide additional guidance on designing future trials .
